# Supplementary material for: Structure and Physical Properties of Mg93−xZnxCa7 Metallic Glasses
Source: Materials (Basel). 2023 Mar 14;16(6):2313. doi: 10.3390/ma16062313 (PMC10057706; doi:10.3390/ma16062313)
Supplement: Supplementary file 1 [file materials-16-02313-s001.zip › materials-2207624-supplementary.pdf]

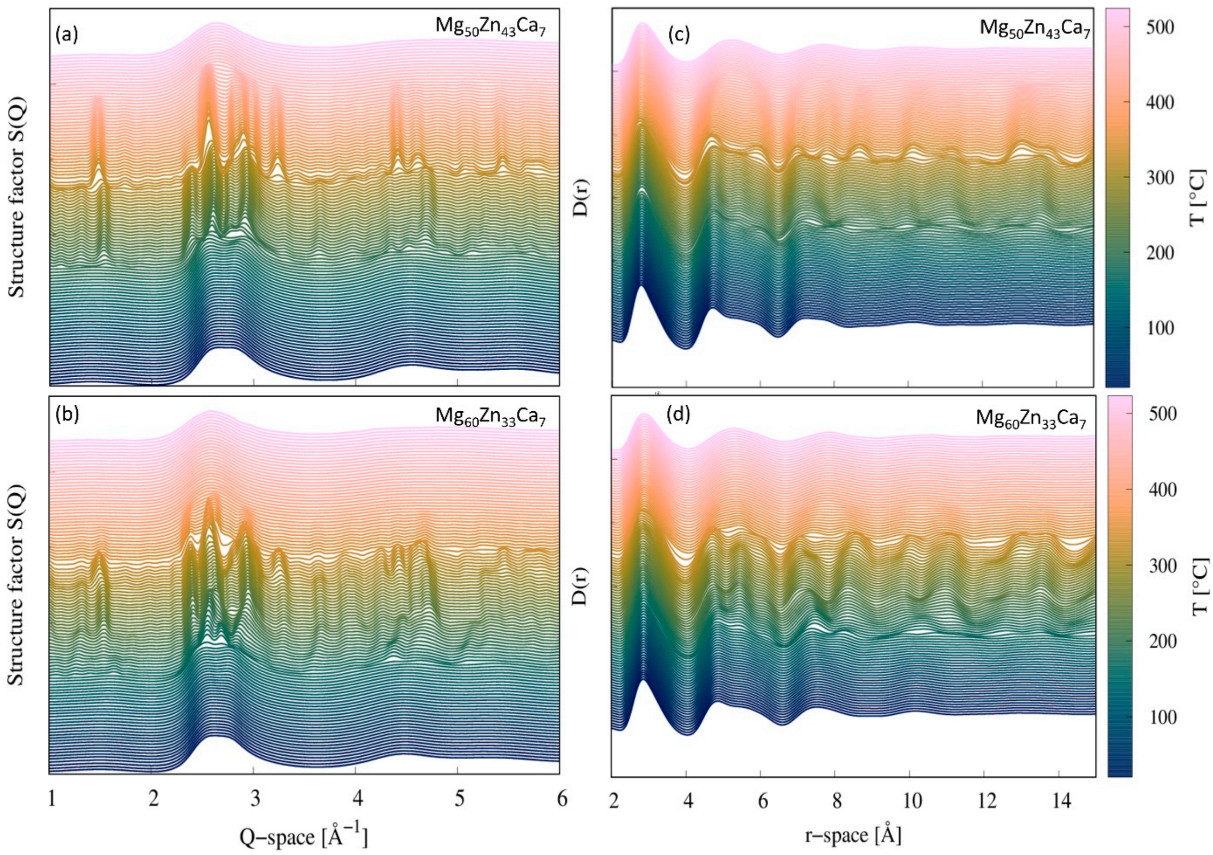

Figure S1. : $S^X(Q)$ and $D^X(r)$ temperature evolution during in situ HEXRD measurements for $Mg_{50}Zn_{43}Ca_7$ (a) and (b) and $Mg_{50}Zn_{43}Ca_7$ (c) and (d).
